# Microbiological Characteristics and Clinical Features of Cardiac Implantable Electronic Device Infections at a Tertiary Hospital in China

**DOI:** 10.3389/fmicb.2017.00360

**Published:** 2017-03-06

**Authors:** Ruobing Wang, Xuebin Li, Qi Wang, Yawei Zhang, Hui Wang

**Affiliations:** ^1^Department of Clinical Laboratory, Peking University People’s HospitalBeijing, China; ^2^Department of Cardiology, Peking University People’s HospitalBeijing, China

**Keywords:** Cardiac implantable electronic device (CIED) infection, pocket, culture, microbial diagnosis, clinical characteristics

## Abstract

The incidence of cardiac implantable electronic device (CIED) infections is rapidly increasing worldwide. However, the microbiological characteristics and clinical features of symptomatic CIED infections are not well described. The present study included patients with CIED infections in China, and their pocket tissues were collected for clinical microbiological determination. A total of 219 patients with CIED infections were investigated; of these patients, 145 (66.2%) were positive for CIED infection in pocket tissue cultures and 24 (11.0%) were positive in both blood and pocket tissue cultures. Patients with recurrent infections and patients with systemic infections tended to have higher rates of positive cultures from pocket tissue. In addition, patients with lung diseases were more likely to have early CIED infections than late CIED infections, while patients with liver diseases were more susceptible to systemic infections than local infections. *Staphylococcus* species were the most common cause of CIED infections; coagulase-negative staphylococci was the predominant type (accounting for 45.2% in all cases and 68.3% in culture-positive cases). None of the *Staphylococcus* isolates were resistant to gentamicin, linezolid or vancomycin. Gram-negative bacilli accounted for 9.1% of all cases and 13.8% of culture-positive cases. Significant differences in the distribution of different pathogens were identified between primary infections and recurrent infections, between local infections and systemic infections, and between early infections and late infections. Our data describe the microbiological characteristics and clinical features of CIED infections, and provide evidence for advisory guidelines on the management of CIED infections in China.

## Introduction

Cardiac implantable electronic device (CIED) infection, is a major complication following implantation of these devices and represents a broad range of symptoms, varying from local complaints at the pulse generator pocket and/or the subcutaneous portion of the leads, to severe systemic manifestations (Tarakji et al., 2010; Sohail et al., 2011; De Bie et al., 2012). Accumulating evidence has suggested an increased rate of CIED infections worldwide due to continuous growth in the utilization of CIEDs (Cabell et al., 2004; Zhan et al., 2008; Mond and Proclemer, 2011). Data from the United States have indicated a 3.1-fold increase in the number of hospitalizations for CIED infection between 1996 and 2003 (Voigt et al., 2006). Patients with CIED infection often have prolonged hospital stay, increased financial burden and higher rates of in-hospital morbidity and mortality. It has been shown that CIED infection increases the risk of in-hospital death by more than twofold (Voigt et al., 2006). In addition, increased mortality persists even after hospital discharge (De Bie et al., 2012), highlighting the need to maximize infection control measures in settings in which CIED infection is likely to occur.

*Staphylococcus* species, including coagulase-negative staphylococci (CoNS), methicillin-sensitive *Staphylococcus aureus* (MSSA), and methicillin-resistant *Staphylococcus aureus* (MRSA), are by far the most common microbiologic causes of CIED infections, accounting for 60%–80% of all CIED infections (Greenspon et al., 2012; Habib et al., 2013; Rickard et al., 2013; Aydin et al., 2016). However, unusual organisms, such as Gram-negative bacilli, are also present in CIED infections. The majority of CIED infections are pocket infections; however, when the infection moves to the intravascular portion of the leads, intravascular infection can occur, resulting in bacteremia and endocarditis. Interestingly, it was shown that the incidence of CIED infection in the setting of *S. aureus* bacteremia could reach 55% (Uslan et al., 2007). In addition, Gram-negative and non-staphylococcal bacteremia can also cause CIED infection, although these infections are usually associated with lower morbidity and mortality (Viola et al., 2010). Therefore, an understanding of the microbiologic risk factors/microbial epidemiology of CIED infections is critical for the selection of both antimicrobial prophylaxis and initial empirical treatments, as the microbiology of CIED infections is closely associated with the pathogenesis of infection.

Two studies have recently described the asymptomatic bacterial colonization of CIEDs in China (Chu et al., 2014; Zou et al., 2014). However, there are limited data regarding the microbiological characteristics and clinical features of symptomatic CIED infections in China. It is of paramount importance to draw a basic picture of the characteristics of symptomatic CIED infections, as this information will provide valuable insights into the pathogenesis, implementation of preventive measures and treatment of CIED infections, especially when considering the rapid increase in methicillin-resistant *Staphylococcus* species in China (Zhao et al., 2012).

## Materials and Methods

### Patients and Case Definitions

From November 2011 to November 2014, a total of 219 consecutive patients with sufficient evidence of CIED infection were included. Patients with CIED infections were defined as the presence of early post-implantation infections; generator pocket infections; constitutional symptoms or CIED-related lead infection or infectious endocarditis (CIED-LI/IE), as previously described (Chinese Heart Rhythm Society, 2013; Sandoe et al., 2015a). Specifically, an early post-implantation infection case was defined as erythema affecting the implantation incision site, without purulent exudate, exposed devices, or systemic signs of infection. Generator pocket infection was defined as the device penetrating through the skin or purulent exudate without systemic signs. Constitutional symptoms included positive blood cultures, and fever or chills without vegetation on the leads or cardiac valves. If vegetation was found by transesophageal echocardiography (TEE), a diagnosis of CIED-LI/IE was considered. Oral anticoagulant treatment was stopped before CIED-related procedures. The demographics and clinical characteristics of all patients with CIED infections are shown in **Table 1**. Overall, patients with hypertension exhibited the highest prevalence of CIED infections (48.4%), followed by patients with diabetes mellitus (24.2%). The incidence of early post-implantation infections, generator pocket infections, constitutional symptoms, and CIED-LI/IE was 14.6% (32/219), 69.4% (152/219), 11.0% (24/219), and 5.0% (11/219), respectively. Positive cultures were identified in 145 patients with CIED infection (24 patients were positive for both blood culture and pocket tissue culture; 121 patients were positive for pocket tissue culture), accounting for 66.2% of all patients. The median length of stay was 15 days, and the in-hospital mortality was 0.9% (2/219). Study protocols were reviewed and approved by the Ethical Committee of Peking University People’s Hospital. Informed consent was not needed as this study was retrospective and participants were anonymized.

**Table 1:** Clinical information and demographics of patients with cardiac implantable electronic device (CIED) infection.

***N* = 219**	
Demographics
Median age at study (max, min), years	68 (58-77)
Male, *n* (%)	161 (73.5)
Median duration of infection (max, min), days	23 (10-37)
Underlying diseases
Lung diseases, *n* (%)	23 (10.5)
Renal diseases, *n* (%)	27 (12.3)
Liver diseases, *n* (%)	24 (11.0)
Diabetes mellitus, *n* (%)	53 (24.2)
Hypertension, *n* (%)	106 (48.4)
Types of infection
Early post-implantation inflammation, *n* (%)	32 (14.6)
Generator pocket infection, *n* (%)	152 (69.4)
Constitutional symptoms, *n* (%)	24 (11.0)
CIED-LI/IE^∗^, *n* (%)	11 (5.0)
Positive culture results
Blood, *n* (%)	24 (11.0)
Pocket tissue, *n* (%)	145 (66.2)
Treatment	
Re-implantation, *n* (%)	137 (62.6)
Inappropriate empirical antimicrobial therapy	68 (31.1)
Definitive antimicrobial therapy^∗∗^, *n* (%)	79 (36.1)
Outcome	
Median length of stay (max, min), days	15 (13-20)
In-hospital death, *n* (%)	2 (0.9)


*^∗^CIED-LI/IE, cardiac implantable electronic device associated lead infection or infectious endocarditis; CoNS, coagulase-negative staphylococci. ^∗∗^Definitive antimicrobial therapy refers to the corrected therapy when microbiology results and antimicrobial susceptibility data were available.*

### Specimen Collection and Culture

Pocket tissue was collected during device extraction. Each specimen (approximately 1 cm^3^ in size) was obtained using an aseptic technique. Pocket tissue samples for culture were placed in sterile bottles and transported to the clinical microbiology laboratory within 12 h. Samples were inoculated onto solid media (blood agar, eosin methylene blue agar, chocolate agar, and Sabouraud agar, Oxoid, Basingstoke, Hampshire, UK). Identification and antimicrobial susceptibility testing were performed using the Vitek 2 automated system (bioMérieux, Marcy-l’Etoile, France). Minimum inhibitory concentrations (MICs) were determined according to Clinical and Laboratory Standards Institute (CLSI) breakpoints (Cockerill, 2011; Patel, 2014).

### Statistical Analysis

The chi-squared or Fisher’s exact test were used for categorical variables. Ordinal and continuous variables were presented as median (interquartile range) and determined by the Mann–Whitney *U* test. A *p*-value less than 0.05 was considered statistically significant. SPSS 19 (SPSS Inc., Chicago, IL, USA) statistical software was used for data analysis.

## Results

### Comparison of Clinical Characteristics of CIED Infections between Patients with Primary Infections and Patients with Recurrent Infections

Of all CIED infections, 112 cases were primary infections and 107 cases were recurrent infections (**Table 2**). Importantly, the incidence of positive cultures from pocket tissue was significantly higher in patients with recurrent infections than in patients with primary infections (*p* = 0.020). A trend of higher incidence of positive cultures from blood was also observed in patients with recurrent infections than in patients with primary infections (*p* = 0.064). Cases for definitive antimicrobial therapy included 45.3% of patients with recurrent infections, which was significantly higher than that in patients with primary infections (*p* = 0.010). Interestingly, the only two cases of in-hospital mortality were observed in patients with recurrent infections. However, no significant difference was observed in the median length of stay between patients with primary infections and patients with recurrent infections (**Table 2**).

**Table 2:** Comparison of clinical characteristics and outcomes between primary and recurrent CIED infections.

Characteristics	Primary infection (*N* = 112)	Recurrence (*N* = 107)	*p*
Infection features			
Median duration of infection occurrence after last procedure (max, min), months	24 (11.35–47)	16 (6–36)	0.171
Systemic infection, *n* (%)	15 (13.4)	21 (19.6)	0.213
Positive culture results			
Pocket tissue, *n* (%)	66 (58.9)	79 (73.8)	0.020^∗^
Blood, *n* (%)	8 (8.4)	16 (19.0)	0.064
Treatment			
Inappropriate empirical antimicrobial therapy, *n* (%)	35 (32.1)	33 (31.1)	0.877
Definitive antimicrobial therapy, *n* (%)	31 (28.4)	48 (45.3)	0.010^∗^
Outcomes			
Median length of stay (max, min), days	15 (13–19.75)	15 (12–20)	0.916
In-hospital death, *n* (%)	0	2 (1.9)	0.146


*^∗^*p* < 0.05.*

### Comparison of Clinical Characteristics between Patients with Early CIED Infections and Patients with Late CIED Infections

Early CIED infection was defined as CIED infection occurring within 6 months of CIED-related procedures (Sandoe et al., 2015a). Overall, 49 patients (22.4%) were identified as having early CIED infection and 170 patients (77.6%) were identified as having late CIED infection (**Table 3**). In particular, patients with lung diseases were more likely to have early CIED infections (OR, 2.857; *p* = 0.016). In addition, patients with diabetes mellitus exhibited a trend of higher incidence of early infections compared to late infections (*p* = 0.052). No significant difference was observed in the median length of stay between patients with early CIED infections and patients with late CIED infections (**Table 3**).

**Table 3:** Comparison of clinical characteristics and outcomes between early and late CIED infections.

Characteristics	Early infection (≤6 months) (*N* = 49)	Late infection (>6 months) (*N* = 170)	*p*	OR (95%CI)
Underlying diseases				
Lung diseases, *n* (%)	10 (20.4)	14 (8.2)	0.016	2.857 (1.180-6.917)
Renal diseases, *n* (%)	5 (10.2)	18 (10.6)	0.938	
Liver diseases, *n* (%)	4 (8.2)	23 (13.5)	0.314	
Diabetes mellitus, *n* (%)	17 (34.7)	36 (21.2)	0.052	
Hypertension, *n* (%)	25 (51.0)	81 (47.6)	0.677	
Positive test results				
TEE^∗^, *n* (%)	4 (8.2)	7 (4.1)	0.253	
Blood culture, *n* (%)	8 (16.3)	16 (9.4)	0.107	
Pocket tissue culture, *n* (%)	35 (71.4)	110 (64.7)	0.381	
Outcomes				
Median length of stay (max, min), days	15 (12-19)	15 (13-20)	0.694	
In-hospital death, *n* (%)	1 (2.0)	1 (0.6)	0.346	


*^∗^TEE, transesophageal echocardiography.*

### Comparison of Clinical Characteristics between Patients with Local CIED Infections and Patients with Systemic CIED Infections

The differences in clinical characteristics between patients with local CIED infections and patients with systemic CIED infections were investigated (**Table 4**). The results showed that patients with liver diseases were more susceptible to systemic infections than local infections (OR, 3.338; *p* = 0.012). In addition, a significantly higher rate of positive cultures from pocket tissue was observed in patients with systemic infections (*p* = 0.006). These patients consistently exhibited a higher rate of definitive antimicrobial therapy (*p* = 0.01). Moreover, the median length of stay was significantly longer in patients with systemic infections compared to patients with local infections (*p <* 0.001).

**Table 4:** Comparison of clinical characteristics, treatment and outcomes between local and systemic CIED infections.

Characteristics	Local infection (*N* = 183)	Systemic infection (*N* = 36)	*p*	OR (95% CI)
Underlying diseases				
Lung diseases, *n* (%)	21 (11.5)	3 (8.3)	0.581	
Renal diseases, *n* (%)	24 (13.1)	3 (8.3)	0.425	
Liver diseases, *n* (%)	15 (8.2)	8 (22.2)	0.012ˆ*	3.338 (1.292–8.626)
Diabetes mellitus, *n* (%)	44 (24.0)	9 (25.0)	0.903	
Hypertension, *n* (%)	93 (50.8)	13 (36.1)	0.106	
Positive pocket tissue culture, *n* (%)	114 (62.3)	31 (86.1)	0.006ˆ*	
Treatment				
Inappropriate empirical antimicrobial therapy, *n* (%)	54 (30.2)	14 (38.9)	0.305	
Definitive antimicrobial therapy, *n* (%)	59 (33.0)	20 (55.6)	0.010ˆ*	
Outcomes				
Median length of stay (max, min), days	15 (12-18)	20.5 (14.25-28)	<0.001ˆ*	
In-hospital death, *n* (%)	2 (1.1)	0	0.529	


*^∗^*p* < 0.05.*

### Microbiological Characteristics of CIED Infections

**FIGURE 1:**
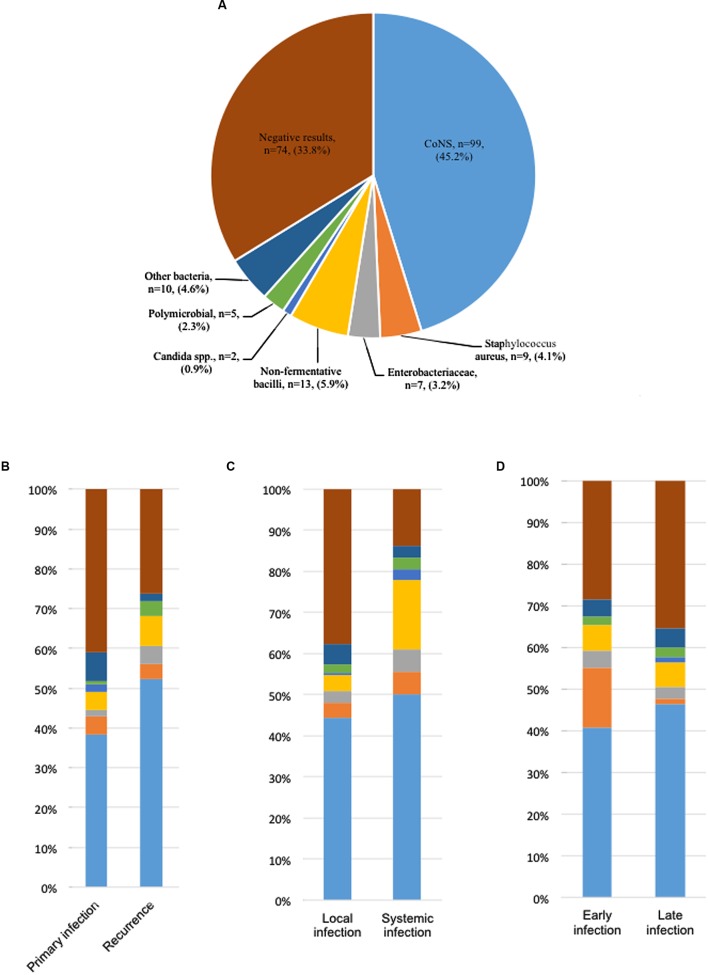
**Pathogen distribution in patients with cardiac implantable electronic device (CIED) infections.** **(A)** Pocket tissue culture results of 219 patients with CEED infections. **(B)** Pathogen distribution in patients with primary and recurrent CIED infections. **(C)** Pathogen distribution in patients with local and systemic CIED infections. **(D)** Pathogen distribution in patients with early and late CIED infections.

In all 145 cases who had a positive culture, 140 cases (96.6%) were monomicrobial infections, and the other five cases were polymicrobial infections (**Figure 1**). The polymicrobials identified in these five cases were *Pseudomonas aeruginosa* and *Klebsiella oxytoca*, *Staphyloccocus epidermidis* and *Staphyloccocus sciuri*, *S. epidermidis* and *Bacillus cereus*, *S. epidermidis* and *Sphingomonas paucimobilis*, and *Enterococcus faecalis* and *S. aureus*, respectively.

Coagulase-negative staphylococci were the most frequently isolated pathogens, accounting for 45.2% of all cases, followed by non-fermentative bacilli (5.9%) and *S. aureus* (4.1%). Gram-negative bacilli were identified in 20 cases, accounting for 9.1% of all cases and 13.8% of culture-positive cases.

The distribution of different pathogens was determined between primary infections and recurrent infections, between local infections and systemic infections, and between early infections and late infections. Importantly, recurrent infections exhibited a higher prevalence of CoNS and non-fermentative bacilli, and a lower prevalence of negative culture and *Candida* compared to primary infections. In addition, non-fermentative bacilli were more easily detected in systemic infections than in local infections (16.7% vs. 3.8%). Furthermore, *S. aureus* was more likely to cause early infections than late infections (**Figure 1**).

### *In vitro* Antimicrobial Susceptibility Testing

*In vitro* antimicrobial susceptibility testing was performed on all culture-positive pathogens. Generally, the prevalence of methicillin resistance among *S. aureus, S. epidermidis*, and remaining CoNS was 11.1, 66.2, and 44.0%, respectively. The resistance rates to erythromycin and clindamycin among these *Staphylococcus* species were 44.4%, 51.4% and 44.0%, 22.2%, 21.6%, and 12.0%, respectively. None of these *Staphylococcus* species were resistant to gentamicin, linezolid or vancomycin (**Table 5**).

**Table 5:** *In vitro* activities of antimicrobial agents against *Staphylococcus* sp..

Antimicrobial agent	*Staphylococcus epidermidis* (*N* = 74)					*Staphylococcus aureus* (*N* = 9)					Remaining coagulase-negative staphylococci (*N* = 25)				
			
	R (%)	I (%)	MIC_50_	MIC_90_	MIC range	R (%)	I (%)	MIC_50_	MIC_90_	MIC range	R (%)	I (%)	MIC_50_	MIC_90_	MIC range
Oxacillin	66.2	0	4	4	0.25–4	11.1	0	0.5	2	0.25–4	44.0	0	0.25	4	0.25–4
Ciprofloxacin	40.5	12.2	2	8	0.5–8	0	11.1	0.5	0.5	0.5–2	8.0	0	0.5	4	0.5–8
Levofloxacin	50.0	4.1	3	8	0.12–8	0	0	0.25	0.25	0.12–1	8.0	16.0	0.25	2	0.12–4
Rifampicin	8.1	0	0.5	1	0.5–32	0	0	0.5	0.5	0.5	8.0	4.0	0.5	2	0.25–4
Gentamicin	0	2.7	0.5	4	0.5–8	0	0	0.5	1	0.5–1	0	4.0	0.5	2	0.5–8
Tetracycline	16.2	4.1	2	16	1–16	11.1	0	1	2	1–16	0	0	1	1	1–2
Clindamycin	21.6	1.4	0.25	8	0.25–8	22.2	0	0.25	8	0.25–8	12.0	4.0	0.25	8	0.25–8
Erythromycin	51.4	2.7	8	8	0.25–8	44.4	0	0.25	8	0.5–8	44.0	8.0	8	8	0.25–8
Linezolid	0	0	1	2	0.25–4	0	0	2	2	1–4	0	0	2	2	0.25–4
Vancomycin	0	0	1	2	0.5–4	0	0	0.5	1	0.5–1	0	0	1	2	0.5–2


*MIC, minimum inhibitory concentration.*

### Comparison of Clinical Characteristics between Gram-Positive Infections and Gram-Negative Infections

The clinical characteristics between Gram-positive infections and Gram-negative infections were assessed (**Table 6**). Of note, in comparison with patients infected by Gram-positive cocci, patients infected by Gram-negative bacilli showed a significantly higher incidence of exposed devices (50.0% vs. 23.2%), fever or chills (25.0% vs. 4.5%), and vegetation (20.0% vs. 5.4%). However, no significant difference in length of stay was noted between patients infected by Gram-positive cocci and patients infected by Gram-negative bacilli. The incidence of antimicrobial adjustment based on the culture results in patients infected by Gram-negative bacilli was 45.0%, which was significantly higher than that in patients infected by Gram-positive cocci (17.0%) (*p* = 0.005).

**Table 6:** Comparison of clinical manifestations, treatment, and outcomes between Gram-positive and Gram-negative pathogens.

	Gram-positive cocci (*N* = 112) (%)	Gram-negative bacilli (*N* = 20) (%)	*P*
Clinical symptoms			
Purulent exudate, *n* (%)	57 (50.9)	8 (40.0)	0.369
Device exposure, *n* (%)	26 (23.2)	10 (50.0)	0.013^∗^
Fever or chills, *n* (%)	5 (4.5)	5 (25.0)	0.001^∗^
Vegetation, *n* (%)	6 (5.4)	4 (20.0)	0.023^∗^
Antimicrobial therapy			
Median duration (max, min), days	13 (9–16)	15.5 (10.5–18.75)	0.174
Adjustment based on culture results, *n* (%)	19 (17.0)	9 (45.0)	0.005^∗^
Inappropriate empirical therapy, *n* (%)	52 (47.7)	10 (50.0)	0.850
Outcomes			
Poor wound healing, *n* (%)	5 (4.5)	2 (10.0)	0.309
Fever or chills, *n* (%)	16 (14.3)	4 (20.0)	0.511
Median length of stay (max, min), days	15 (13–19)	16.5 (14–22.5)	0.199
In-hospital death, *n* (%)	0	0	–


*^∗^*p* < 0.05.*

## Discussion

In this study, we comprehensively analyzed the microbiological characteristics and clinical features of CIED infections in China. To our knowledge, this is the first and the largest study regarding CIED infections in China. The major findings in this study are as follows: (1), patients with recurrent infections and patients with systemic infections tended to have higher rates of positive cultures from pocket tissue; (2), patients with lung diseases were more likely to have early CIED infections than late CIED infections; (3), patients with liver diseases were more susceptible to systemic infections than local infections; (4), *Staphylococcus* species were the most common cause of CIED infections with CoNS the predominant type; Gram-negative bacilli accounted for 9.1% of all cases and 13.8% of culture-positive cases; (5), significant differences in the distribution of different pathogens existed between primary infections and recurrent infections, between local infections and systemic infections, and between early infections and late infections; and 6), none of the *Staphylococcus* isolates were resistant to gentamicin, linezolid or vancomycin.

In this study, CoNS, which are part of the normal skin flora, were responsible for the majority of CIED infections (45.2% in all cases and 68.3% in culture-positive cases), indicating that most CIED infections were introduced at the time of implantation, as suggested by other studies (Bongiorni et al., 2012; Emilie et al., 2012; Nagpal et al., 2012). Interestingly, infections caused by *S. aureus* were only detected in 4.1% of all cases and in 6.2% of culture-positive cases. In addition, Gram-negative bacterial infections were identified in 9.1% of all cases and in 13.8% of culture-positive cases. A retrospective study of 189 cases of CIED infections at a single center in the United Stated revealed that CoNS and Gram-negative bacilli accounted for 42 and 9% of all infections, respectively, which were similar to our findings (Sohail et al., 2007). However, in the United States study, *S. aureus* was responsible for another 29% of infections, which was much higher than that in our study (29% vs. 4.1%) (Sohail et al., 2007). Of note, another study from Italy reported that CoNS, *S. aureus*, and Gram-negative bacilli were detected in 69.0, 13.8, and 6.2% of all culture-positive cases, respectively (Bongiorni et al., 2012). In addition, a higher percentage of *S. aureus* was noted compared to our study (13.8% vs. 6.1%). The high percentage of CoNS and low percentage of *S. aureus* observed in our study may indicate the involvement of host- or device-specific factors that favor infection by CoNS over *S. aureus*. It has been demonstrated that CoNS, compared with *S. aureus*, are likely to be introduced onto device surfaces during insertion (Otto, 2009). In addition, CoNS have been shown to be more adept at evading the host innate immune system than *S. aureus* by being resistant to phagocytosis by neutrophils (Guenther et al., 2009).

A number of associations between clinical characteristics and different types of CIED infections were identified in our study. Interestingly, previous studies have suggested that renal insufficiency and diabetes mellitus are important risk factors for CIED infections (Bloom et al., 2006). In our study, we observed that 12.3 and 24.2% of patients with CIED infections had renal diseases and diabetes mellitus, respectively. In addition, patients with lung diseases were more likely to have early CIED infections. These data indicate that patients with these underlying conditions should be closely monitored after they receive a CIED. Moreover, we found that patients with liver diseases had a higher chance of systemic infections, suggesting that measures should be taken to avoid systemic infections in these patients.

As our study suggests that most CIED infections were introduced at the time of implantation, we recommend the use of antimicrobial prophylaxis which covers *Staphylococcus* species immediately before device insertion, as suggested by other studies (De Oliveira et al., 2009; Sandoe et al., 2015b). It is noteworthy that the majority of *S. epidermidis* isolates were resistant to methicillin. As *S. epidermidis* was the predominant pathogen causing CIED infections in our study, the clinical relevance of our findings indicate that vancomycin should be used as first-line empirical therapy, as suggested by other studies (Emilie et al., 2012). In addition, based on our *in vitro* susceptibility test results, other antimicrobials, including rifampicin, gentamicin and linezolid, may be considered as alternatives for empirical therapy, particularly when vancomycin cannot be tolerated. However, it should be noted that when culture and *in vitro* susceptibility results become available, antimicrobial therapy should be adjusted accordingly.

In summary, our data describe the microbiological characteristics and clinical features of CIED infections, and provide evidence for advisory guidelines on the management of CIED infections in China.

## Author Contributions

RW and HW conceived and designed the research and interpreted the data. RW and QW contributed to acquisition and analysis of data. RW and YZ drafted the manuscript. XL and HW contributed to the conception of the work and did final approval of the version to be published.

## Conflict of Interest Statement

The authors declare that the research was conducted in the absence of any commercial or financial relationships that could be construed as a potential conflict of interest.
