# Corrigendum: Microbiological Characteristics and Clinical Features of Cardiac Implantable Electronic Device Infections at a Tertiary Hospital in China

**DOI:** 10.3389/fmicb.2017.00928

**Published:** 2017-05-19

**Authors:** Ruobing Wang, Xuebin Li, Qi Wang, Yawei Zhang, Hui Wang

**Affiliations:** ^1^Department of Clinical Laboratory, Peking University People's HospitalBeijing, China; ^2^Department of Cardiology, Peking University People's HospitalBeijing, China

**Keywords:** cardiac implantable electronic device (CIED) infection, pocket, culture, microbial diagnosis, clinical characteristics

In the original article, we neglected to thank the funder National Natural Science Foundation of China, 81625014 to Hui Wang. The authors apologize for this error and state that this does not change the scientific conclusions of the article in any way.

## Conflict of interest statement

The authors declare that the research was conducted in the absence of any commercial or financial relationships that could be construed as a potential conflict of interest.

